# Molecular cytogenetic identification of three rust-resistant wheat-*Thinopyrum ponticum* partial amphiploids

**DOI:** 10.1186/s13039-018-0378-0

**Published:** 2018-05-02

**Authors:** Yanru Pei, Yu Cui, Yanping Zhang, Honggang Wang, Yinguang Bao, Xingfeng Li

**Affiliations:** 1 State Key Laboratory of Crop Biology, Shandong Agriculture University, Tai’an, 271018 Shandong China; 2 College of Agronomy, Shandong Agriculture University, Tai’an, 271018 Shandong China

**Keywords:** *Thinopyrum ponticum*, Common wheat, Partial amphiploids, In situ hybridization, Stripe rust, Leaf rust

## Abstract

**Background:**

*Thinopyrum ponticum* (2n = 10× = 70, J^S^J^S^J^S^J^S^JJJJJJ) is an important wild perennial *Triticeae* species that has a unique gene pool with many desirable traits for common wheat. The partial amphiploids derived from wheat-*Th. ponticum* set up a bridge for transferring valuable genes from *Th. ponticum* into common wheat.

**Results:**

In this study, genomic in situ hybridization (GISH), multicolor GISH (mcGISH) and fluorescence in situ hybridization (FISH) were used to analyze the genomic constitution of SN0389, SN0398 and SN0406, three octoploid accessions with good resistance to rust. The results demonstrated that the three octoploids possessed 42 wheat chromosomes, while SN0389 contained 12 *Th. ponticum* chromosomes and SN0398 and SN0406 contained 14 *Th. ponticum* chromosomes. The genomic constitution of SN0389 was 42 W + 12J^S^, and for SN0398 and SN0406 it was 42 W + 12J^S^ + 2 J. Chromosomal variation was found in chromosomes 1A, 3A, 6A, 2B, 5B, 6B, 7B, 1D and 5D of SN0389, SN0398 and SN0406 based on the FISH and McGISH pattern. A resistance evaluation showed that SN0389, SN0398 and SN0406 possessed good resistance to stripe and leaf rust at the seedling stage and adult-plant stage.

**Conclusions:**

The results indicated that these wheat-*Th. ponticum* partial amphiploids are new resistant germplasms for wheat improvement.

**Electronic supplementary material:**

The online version of this article (10.1186/s13039-018-0378-0) contains supplementary material, which is available to authorized users.

## Background

*Thinopyrum ponticum* (Podp.) Barkworth & D.R. Dewey [syn. *Agropyron elongatum* (Host) P. Beauv., *Lophopyrum ponticum* (Popd.) A. Löve, *Elytrigia pontica* (Popd.) Holub] (2n = 10× = 70), a perennial Triticeae species that is closely related to wheat, has been used for more than half a century to enrich the wheat germplasm with desirable traits [3]. Many important genes have been successfully transferred to common wheat from *Th. ponticum*, including resistance to powdery mildew [18], stripe rust [12, 30], leaf rust [24], stem rust [7, 23], Fusarium head blight [9, 14, 26, 27] and wheat streak mosaic virus [17], as well as abiotic stress tolerance [4, 28], and even yield-related traits [16, 21]. Although it is widely used in wheat improvement, the genomic composition of *Th. ponticum* has been long debated. Past research suggests that *Th. ponticum* is a decaploid with the genome formula JJJJJJJJJJ [22]. Using St genomic DNA from the diploid *Pseudoroegneria strigosa* as a probe and the E genomic DNA from *Th. elongatum* for blocking, Chen et al. [3] revealed that the genomic composition of *Th. ponticum* was J^S^J^S^J^S^J^S^JJJJJJ. The J genome of *Th. ponticum* is homologous to the J genome of the diploid *Thinopyrum bessarabicum*, while the J^S^ genome is a modified J genome of unknown origin [3].

Stripe rust and leaf rust are both severe foliar diseases in wheat (*Triticum aestivum* L.) all over the world. Stripe rust is caused by the fungus *Puccinia striiformis* f.sp*. Tritici*., and it can cause severe yield loss in common wheat [25]. Leaf rust is caused by *Puccinia recondita f. sp. Tritici.*, which is the most widespread and regularly occurring rust on wheat and can also cause yield losses up to 50% in extremely susceptible cultivars [6]. Breeding resistant cultivars is the most effective and economical means to control the disease [13]. At the present time, many of the existing resistance genes have been overcome by newly emerged virulent isolates. Thus, it is necessary and pressing to exploit new resistant genes for wheat breeding.

Genomic in situ hybridization (GISH) is widely used and is an effective means for detecting alien chromosomes and chromosome segments in wheat-alien species amphiploids, addition lines, and translocation lines. Multicolor GISH (mcGISH) is used to discriminate the A, B, D and E genomes of wheat - *Th. ponticum* addition, substitution and translocation lines [9, 10]. Fluorescence in situ hybridization (FISH), which uses repetitive DNA clones or oligonucleotides as a probe, is an extremely useful method for identifying chromosomes within a species or detecting intergenomic chromosome rearrangements in a polyploid species [5, 15, 19].

In this study, three novel wheat-*Th. ponticum* partial amphiploids were developed from derivatives of common wheat and *Th. ponticum*, and FISH, GISH and mcGISH analyses were used to identify their genomic constitution. Furthermore, the resistance to stripe and leaf rust of the three partial amphiploids was also identified.

## Methods

### Plant materials

The plant materials used in this study included *Th. ponticum, Pseudoroegneria spicata* (StSt, 2n = 14), *Aegilops speltoides* (SS, 2n = 14), *Aegilops tauschii* (DD, 2n = 14), the common wheat cultivar Yannong15 (YN15) and three wheat-*Thinopyrum ponticum* partial amphiploids (SN0389, SN0398 and SN0406). Among them, *Th. ponticum* was provided by Prof. Zhensheng Li (formerly of the Northwest Institute of Botany at the Chinese Academy of Sciences, Yangling, China). *Ps. spicate, Triticum urartu*, *A. speltoides* and *A. tauschii* were provided by Prof. Lihui Li from the Institute of Crop Science, Chinese Academy of Agricultural Sciences, Beijing, China. The partial amphiploids SN0389, SN0398 and SN0406 were selected from BC_1_F_7_ of common wheat Yannong15 crossed with *Th. ponticum*, based on the stability and good phenotypic characteristics, such as long spikes, advanced fluorescence, and so on. The amphiploids were maintained by selfing in our laboratory.

### Mitotic and meiotic studies

The seeds were germinated at 25 °C on moistened filter paper in petri dishes for 24 h, were maintained at 4 °C for approximately 1 day, and were then transferred to 25 °C for approximately 12 h. Roots, of a length of 1–2 cm, were collected and immediately placed in ice water. After 24–32 h, these roots were fixed in Carnoy’s solution for 24 h and were then stored in 70% (*v*/v) ethanol. The root tips were squashed in acetic acid and were observed under a phase contrast microscope. When the flag leaf of the wheat was spread, the young spikes were sampled, and the anthers, at metaphase I (MI) of meiosis, were fixed in Carnoy’s solution, dissociated in 1 M HCl at 60 °C for 6–8 min, and homogenized in 1% acetocarmine.

### Genomic in situ hybridization (GISH)

Genomic DNA from *Ps. spicata* was labeled with Texas red-5-dCTP by the nick translation method and was used as a probe. Sheared genomic DNA from Yannong15 was used as the blocking DNA. The slides were counterstained with DAPI in Vectashield mounting medium (Vector Laboratories, USA). The detailed procedures of the chromosome spread preparation and hybridization are described by Bao et al. [1, 2]. The J^S^-genomic and J-genomic chromosomes were distinguished by the GISH signals [3, 29], and those with centromeres labeled by the red signals were the J^S^-genome and those with two arm ends of chromosomes labeled by signals were the J-genome.

### Multicolor genomic in situ hybridization (mcGISH)

Total genomic DNA from *T. urartu*, *A. speltoides* and *A. tauschii* was isolated from the young leaves via a modified CTAB method. The total genomic DNA from *T. urartu* was labeled with fluorescein-12-dUTP, and the genomic DNA from *A. tauschii* was labeled with Texas-red-5-dUCP by the nick translation method. Total genomic DNA from *A. speltoides* was used a blocker (at a ratio of 1:160). After hybridization, the slides were washed in 2× saline sodium citrate (SSC) and mounted in Vectashield mounting medium.

### Fluorescence in situ hybridization (FISH)

Two probes were used in the multicolor FISH. pAs1 was labeled with fluorescein-12-dUTP, and the repeated DNA sequence, (GAA)_8_, was labeled with Texas-red-5-dUCP. Before hybridization, the two probes were mixed at a ratio of 4:1. The detailed procedures for the hybridization were previously described by He et al. [11]. Images were captured with an Olympus BX-60 fluorescence microscope equipped with a CCD (charge-coupled device) camera.

### Stripe rust and leaf rust resistance evaluation

The stripe rust resistance of the three partial amphiploids, at the seedling stage, was evaluated with stripe rust race CYR32 in a greenhouse that had a favorable environment for stripe rust development at the Shandong Academy of Agricultural Sciences, Jinan, China. At the adult-plant stage, stripe rust and leaf rust resistance were evaluated under natural conditions. YN15 (the susceptible cultivar for stripe rust and leaf rust) and *Th. ponticum* were planted as contrasts at the same time. When the control variety YN15 was all fully infected, the evaluation results were scored according to the standard classification system with 6 scales from 0 to 4 as follows: 0 for no visible symptoms; 0, for necrotic flecks without sporulation; and 1, 2, 3, and 4 for strongly resistant, resistant, susceptible and strongly susceptible, respectively.

## Results

### Chromosomal constitution of three partial amphiploids

**Fig. 1:**
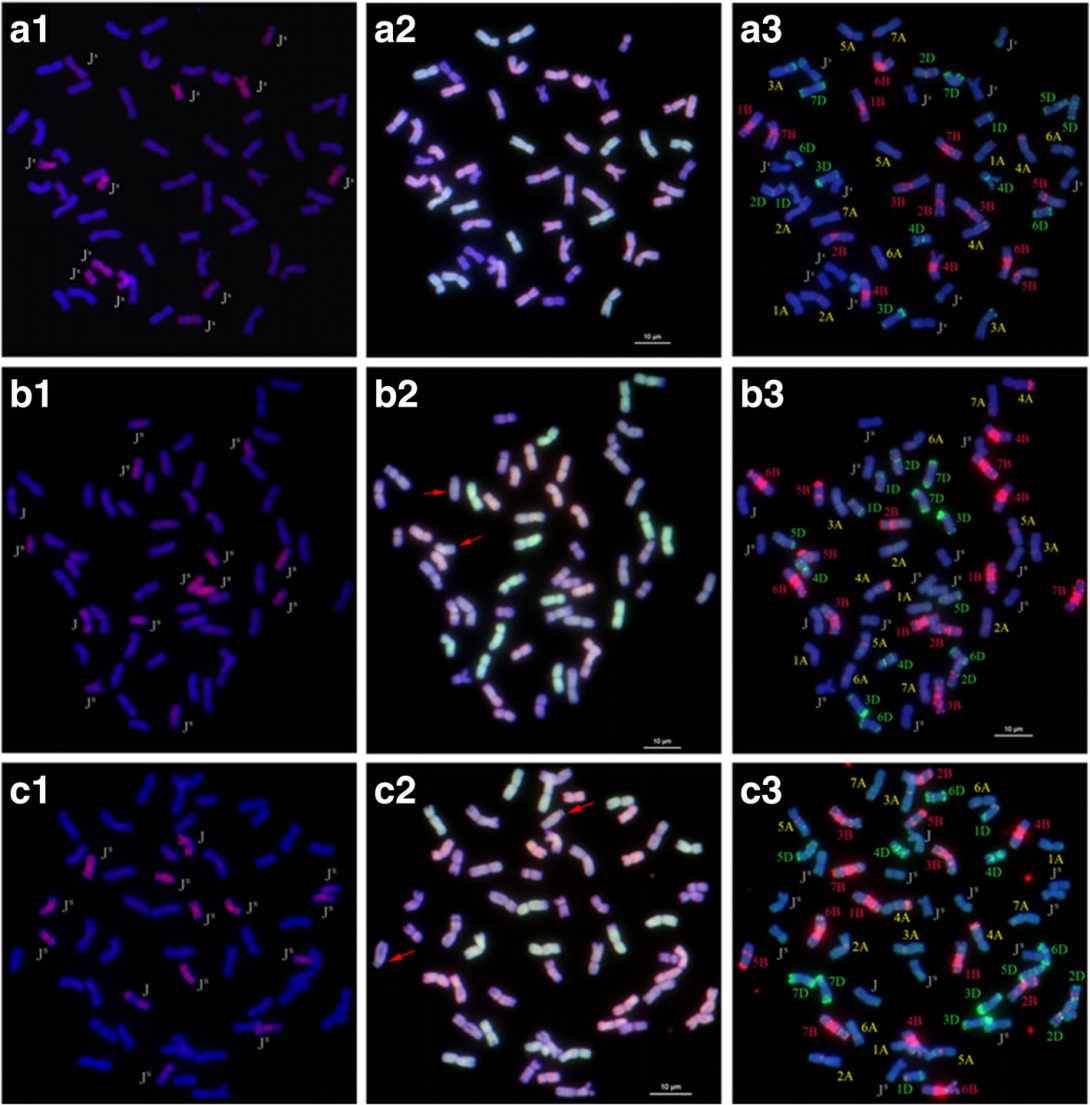
GISH, McGISH and FISH patterns of SN0389, SN0398 and SN0406. GISH patterns of SN0389 (**a1**), SN0398 (**b1**) and SN0406 (**c1**): *Ps*. *spicata* (St) genomic DNA labeled with Texas-Red-5-dCTP was used as the probe, and YN15 genome DNA was used to block. McGISH patterns of SN0389 (**a2**), SN0398 (**b2**) and SN0406 (**c2**): A-genomic DNA was labeled with green fluorescence, D-genomic DNA was labeled with red fluorescence and B-genomic DNA (gray) was used to block. As a result, *Th. ponticum* chromosomes showed purple signals. The FISH patterns of SN0389 (**a3**), SN0398 (**b3**) and SN0406 (**c3**): red signals were (GAA)_8_ and green signals were pAs1

An analysis of the mitotic chromosomes showed that SN0389 contained a chromosome number of 2n = 54, and both SN0398 and SN0406 had a mitotic chromosome number of 2n = 56 (Fig. 1). The meiotic observations of the three partial amphiploids indicated that most of the chromosomes in the observed pollen mother cells of SN0389 formed 27 bivalents at meiotic MI, and SN0398 and SN0406 both formed into 28 bivalents, which proved that these three partial amphiploids exhibited high cytological stability.

**Fig. 2:**
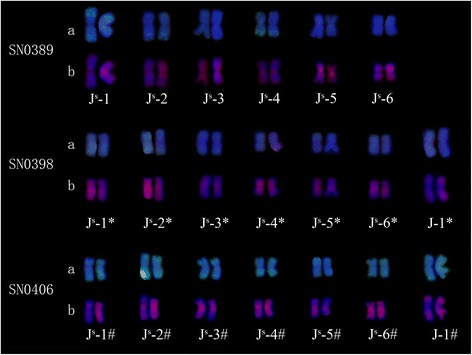
FISH and GISH patterns of the alien chromosomes of SN0389, SN0398 and SN0406. a. FISH patterns of the alien chromosomes; b. GISH patterns of the alien chromosome. * behind the chromosome numbers indicate the J^S^ and J chromosomes in SN0398. # behind the chromosome numbers indicate the J^S^ and J chromosomes in SN0406. The alien chromosomes in SN0389, SN0398 and SN0406 with the same number did not mean the same chromosomes

GISH, mcGISH and FISH were used to analyze the genomic constitution of SN0389, SN0398 and SN0406. The results of the GISH (Fig. 1-A1) and FISH (Fig. 1-A3) analyses revealed that SN0389 had 42 wheat chromosomes and 12 *Th. ponticum* chromosomes, including six pairs of J^S^-genome chromosomes (Fig. 2). SN0398 contained 42 wheat chromosomes and 14 *Th. ponticum* chromosomes (Fig. 1-B1, B2, B3), including six pairs of J^S^-genome chromosomes and one pair of J-genome chromosomes (Fig. 2). The genomic constitution of SN0406 (Fig. 1-C1, C2, C3 and Fig. 2) was similar to SN0398. According to the configuration and signal of the alien chromosomes, the alien chromosomes of SN0389 were different compared to SN0398 and SN0406, while some of the alien chromosomes in SN0398 were probably identical to that in SN0406. For example, the J^S^-4*, J^S^-6* and J-1* chromosomes in SN0398 were similar with the J^S^-4^#^, J^S^-6^#^ and J-1^#^ chromosomes in SN0406 (Fig. 2). But as *Th. ponticum* contained 14 pairs of J^S^ and 21 pairs of J chromosomes, and there were lack of specific in situ hybridization signals and specific molecular markers of each pairs of chromosomes in *Th. ponticum*, so it’s difficult to identify the alien chromosomes in these three amphiploids.

### Wheat chromosome variation of three partial amphiploids

**Fig. 3:**
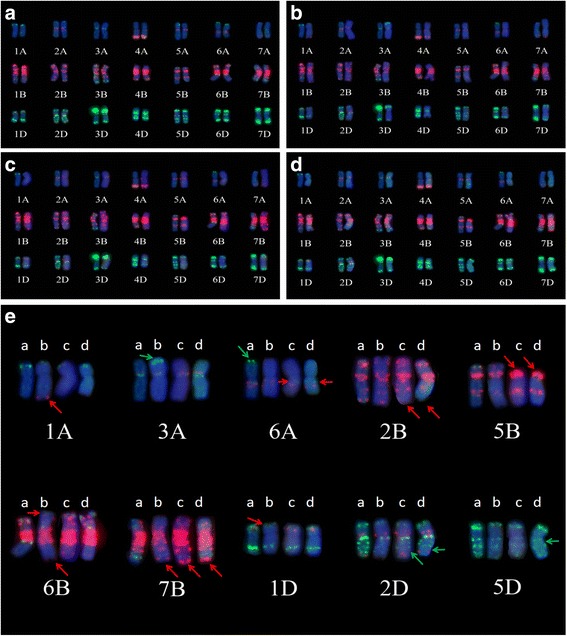
FISH patterns of the wheat chromosomes in SN0389, SN0398 and SN0406 compared with the common wheat YN15. FISH patterns of YN15 (**a**), SN0389 (**b**), SN0398 (**c**), and SN0406 (**d**). In chromosomes H, I and J, on the left, were those in YN15, and those on the right were from the partial amphiploids. **e**, chromosome variations between YN15 and three partial amphiploids based on the FISH patterns. Among them, *a*, *b*, *c* and *d* indicated the corresponding chromosome of YN15, SN0389, SN0398 and SN0406 in turn

The wheat chromosome variation in the three partial amphiploids was analyzed using FISH and McGISH signals, and the results of the common wheat parent YN15 were used as a comparison (Fig. 3). For the A-genome chromosomes, the results showed that the additional red (GAA)_8_ signals were detected at the terminal of 1AL in SN0389 (Fig. 3-E). Additional, apparently green, signals of the pAs1 probe were also found on 3AS of SN0389. Moreover, the green signal present in the terminal of 6AS in YN15 disappeared in the three partial amphiploids, and a pair of (GAA)_8_ signals of 6AL in SN0398 and SN0406 was absent (Fig. 3-E).

The red signals of (GAA)_8_ in the B-genome chromosomes of SN0389, SN0398 and SN0406 were also changed a lot. For example, the (GAA)_8_ signals at the long arm of 2B were absent in SN0398 and SN0406, while the red signal still remained in SN0389, and the absence was also observed on the satellite of 6B in SN0389. Additional red signals were observed on 7BL of the three partial amphiploids. Furthermore, the (GAA)_8_ signal on the terminal of 6BL in SN0389 was different from the other three materials and approached the end of 6BL in SN0389. Moreover, part of the 5B short arm in SN0398 and SN0406 was absent, compared with that of YN15 and SN0389. A few green signals of pAs1 were present on the 6BL chromosomes in YN15, while they were not observed in the three germplasms (Fig. 3-E).

For the D-genome chromosomes in SN0389, SN0398 and SN0406, variations occurred in the 1D, 2D and 5D chromosomes. First, the red signals of (GAA)_8_ on 1DS were absent in SN0389, while they were preserved in YN15, SN0398 and SN0406. Additionally, the green signals of pAs1, near the terminal of 2DL, were also absent in SN0398 and SN0406. Moreover, the pAs1 signal near the centromere of the 5D chromosomes in SN0389 and SN0398 was absent (Fig. 3-E).

### Phenotypic evaluation of three partial amphiploids

The stripe rust resistance of SN0389, SN0398 and SN0406 at the seedling stage was evaluated in a greenhouse, while stripe and leaf rust resistance was evaluated under natural conditions (Table 1, Additional file 1: Figure S1). The results showed that SN0389, SN0398 and SN0406 showed good resistance to stripe rust race CYR32 in the seedling stage. At the adult-plant stage, these three partial amphiploids were immune to stripe rust and showed good resistance to leaf rust in the field. While its common wheat parent YN15 was susceptible to stripe rust and leaf rust and *Th*. *ponticum* was immune, we deduced that the resistance of partial amphiploids was derived from *Th*. *ponticum*.

**Table 1:** Stripe rust and leaf resistance evaluation of SN0389, SN0398 and SN0406

Materials	Stripe rust CYR32	Stripe rust	Leaf rust
	Seedling stage	Adult-plant stage	Adult-plant stage
*Th*. *ponticum*	0	0	0
YN15	4	4	4
SN0389	0;	0	0;
SN0398	0;	0	0;
SN0406	0;	0	0;

## Discussion

Although *Th*. *ponticum* is closely related to wheat, it is difficult to obtain excellent germplasm materials directly by the hybridization between *Th*. *ponticum* and common wheat. Thus, wheat-*Th. ponticum* partial amphiploids, which contain the complete genomes of wheat but an incomplete genome (a set of chromosomes) of *Th*. *ponticum*, are used as crucial intermediate materials in the transfer of desirable genes from *Th*. *ponticum* to common wheat [3]. Several wheat-*Th. ponticum* amphiploids have been obtained, analyzed and exploited as alien sources of disease resistance in wheat improvement [3, 8, 12, 24, 31].

In this study, we identified three novel wheat-*Th. ponticum* partial amphiploids with good rust resistance indpendent of stage. These three octoploid *Trititrigia* were developed in the common wheat cultivar YN15 background, and they had good phenotypic characteristics, such as long spikes, advanced fluorescence, and higher cross-compatibility with wheat. Therefore, they could be used as bridge parents to cross with wheat to develop addition, substitution or translocation lines in order to provide new rust resistance germplasms for wheat breeding.

At both stages, YN15 was highly susceptible to stripe rust and leaf rust, whereas the three octoploid *Trititrigia* were immune at both stages. This indicated that SN0389, SN0398 and SN0406 possessed a resistant gene to rust that was derived from *Th. ponticum*. An analysis of the mitotic chromosomes showed that SN0389 had 42 wheat chromosomes and 12 *Th. ponticum* chromosomes, while SN0398 and SN0406 had 42 wheat chromosomes and 14 *Th. ponticum* chromosomes. The results indicated that the alien chromosome of SN0389 was 12J^S^. The alien chromosomes in SN0389 and SN0406 were not from a single genome of J^S^ or J, and the alien chromosome constitution of SN0398 and SN0406 was 12J^S^ + 2 J. Since there were 12 or 14 alien chromosomes in these three octoploid *Trititrigia*, it was difficult to deduce which chromosome the rust resistance gene was located on. We had hybridized the amphiploid with YN15, thus try to screen addition lines with different alien chromosomes in the derivative generations. Then it will be easier to identify which alien chromosome carrying the rust resistance gene. As there were lack of specific FISH or GISH signals and specific molecular markers of each chromosomes in *Th. ponticum* now, more work is needed to make that conclusion.

It is generally believed that only euploid amphiploids are genetically stable, while aneuploids often result in the loss of the added alien chromosomes [20]. Nevertheless, our results here show that the chromosome number of SN0389 was 2n = 54, and it only contained 12 alien chromosomes. However, the meiotic studies showed that SN0389 had a regular meiotic behavior after it was self-pollinated for several generations, and its chromosome number was still 2n = 54. Similar phenomena are also observed in partial amphiploid lines obtained from wheat × *Th. ponticum* and wheat × *Th. intermedium* hybridizations [8, 10, 24]. For example, the partial amphiploid BE-1 contains 16 chromosomes derived from *Th. ponticum* and 40 wheat chromosomes, and the substituted wheat chromosome pair, as identified by FISH, was 7D [24]. Lines Zhong 1 (2n = 52) and Zhong 2 (2n = 54) both contain the complete wheat A, B and D genomes but with 10 and 12 *Th. intermedium* chromosomes, respectively [10]. Further research on the composition of these alien chromosomes and its compensation effect will be helpful in understanding the genetic relationship between the genome of *Th. ponticum* and wheat as well as be a benefit for transferring valuable traits from *Th*. *ponticum* into wheat.

Using the in situ hybridization pattern of FISH and McGISH, chromosome variations in wheat were also detected. In this study, the structural variations also occurred in chromosomes 1A, 3A, 6A, 2B, 5B, 6B, 7B, 1D and 5D of SN0389, SN0398 and SN0406. The results indicated that during the formation of the partial amphiploids, various intergenomic rearrangements occurred. Some of the chromosome recombinations were caused by introgressed chromosome segments from *Th. ponticum* into common wheat chromosomes, while the introgressed segments were too small to detect by GISH. The other reason for the structural variations in the wheat chromosomes might also be that they were generated by recombination between different wheat chromosomes, such as homeologous chromosome recombination between the A-, B-, and D- genome genomic chromosomes that was interfered by the existence of the *Th. ponticum* chromosomes.

## Conclusions

Three partial amphiploids with good resistance and different phenotypic traits were obtained in this study. The chromosome composition of the wheat-*Th ponticum* partial amphiploid was studied by means of GISH, McGISH and FISH. As a good source for improving disease resistance, these amphiploids could be used as promising crossing partners in wheat breeding programs, and resistant progenies of this partial amphiploid could be used as rust resistance sources in wheat improvement.

## Additional file

Additional file 1: **Figure S1.** Reaction to stripe rust CYR32 of SN0389, SN0398 and SN0406. (TIF 1679 kb)

## Abbreviations

FISH: Fluorescence in situ hybridization
GISH: In situ hybridization
mcGISH: Multicolor GISH
MI: Metaphase I
