# Supplementary figures and images for: Molecular cytogenetic identification of three rust-resistant wheat-Thinopyrum ponticum partial amphiploids
Source: Mol Cytogenet. 2018 May 2;11:27. doi: 10.1186/s13039-018-0378-0 (PMC5930962; doi:10.1186/s13039-018-0378-0)

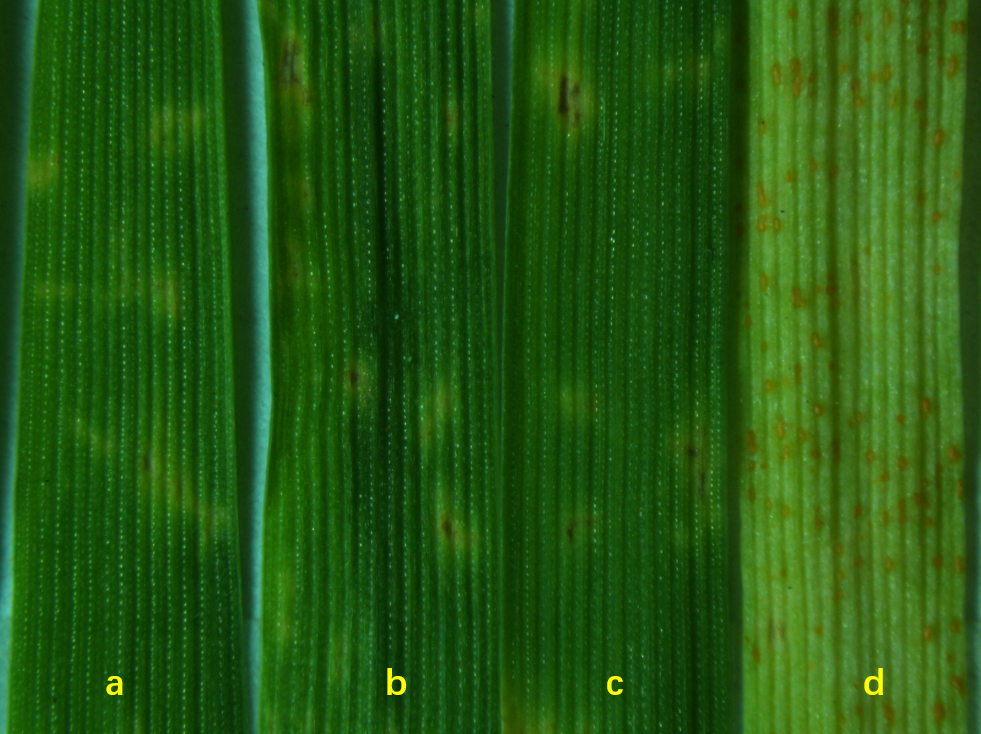

Supplement: Supplementary file 1 — Figure S1. Reaction to stripe rust CYR32 of SN0389, SN0398 and SN0406. (TIF 1679 kb) [file 13039_2018_378_MOESM1_ESM.tif]
